# Relationship between Microbial Translocation and Endothelial Function in HIV Infected Patients

**DOI:** 10.1371/journal.pone.0042624

**Published:** 2012-08-30

**Authors:** Emily Blodget, Changyu Shen, Grace Aldrovandi, Adrienne Rollie, Samir K. Gupta, James H. Stein, Michael P. Dubé

**Affiliations:** 1 Department of Medicine, Division of Infectious Diseases, University of Southern California Keck School of Medicine, Los Angeles, California, United States of America; 2 University of Southern California Keck School of Medicine, Los Angeles, California, United States of America; 3 Department of Biostatistics3 Indiana University School of Medicine, Indianapolis, Indiana, United States of America; 4 Department of Infectious Diseases, Indiana University School of Medicine5, Indianapolis, Indiana, United States of America; 5 Indiana University School of Medicine, Indianapolis, Indiana, United States of America; 6 Department of Pediatrics, Children's Hospital of Los Angeles, Los Angeles, California, United States of America; 7 Division of Cardiovascular Medicine, University of Wisconsin School of Medicine and Public Health, Madison Wisconsin, United States of America; University of Nebraska Medical Center, United States of America

## Abstract

**Background:**

Circulating levels of microbial products are increased in HIV infection, and provoke endothelial dysfunction in other disease settings.

**Methodology/Principal Findings:**

We examined data from a cross-sectional single site study at Indiana University (Indiana, N = 85) and a 24- week multicenter prospective study of antiretroviral therapy (ART) initiation (ACTG 5152s, N = 75). Brachial artery flow-mediated dilation (FMD) was measured by ultrasound. Plasma lipopolysaccharide (LPS) and soluble CD14 (sCD14) levels were measured from stored specimens and correlated with FMD values using Pearson correlations. The Indiana subjects were 63% male with a mean age of 39 years and a median CD4 count of 406 cells/mm^3^ (388 not on ART, 464 on ART). The 5152s subjects were 92% were male with a mean age of 35 years and a median CD4 count of 251 cells/mm^3^ at entry which increased to 396 cells/mm^3^ on ART. When analyzing the two cohorts individually or in combination neither sCD14 nor LPS correlated significantly with FMD. In a pre-specified subgroup analysis of the Indiana subjects receiving ART (N = 46, mean ART duration 40 months) LPS was inversely correlated with FMD (r = −0.33, p = 0.02), but not sCD14 (r = −0.01, p = 0.9). Multivariate analysis confirmed LPS as an independent predictor of FMD in this subgroup (p = 0.02).

**Conclusions/Significance:**

**I**n HIV- infected individuals on prolonged ART, higher LPS levels are associated with worse endothelial function but not in untreated subjects or at 24 weeks after ART initiation. Persistent microbial translocation may contribute to arterial dysfunction and the increased cardiovascular disease risk observed in individuals on long-term ART.

## Introduction

HIV infection has been associated with increased cardiovascular disease risk [1] but the mediators of the increased risk have not been specifically identified. Endothelial dysfunction is a critical initial step of atherogenesis which subsequently contributes to the progression and clinical manifestations of atherosclerosis [2], [3]. Long-term use of protease inhibitors (PIs) has been associated with endothelial dysfunction [4]. Factors other than PI use that may contribute to endothelial dysfunction in HIV-infected patients include untreated HIV infection itself [5] treatment-associated lipid changes [4], [6] and the lipodystrophy syndrome [7].Mediators and markers of endothelial dysfunction have been sought, such as lipids and lipoproteins and circulating markers of inflammation and vascular activation, but the majority of these factors have not been significantly associated with endothelial function as measured by brachial flow-mediated dilation (FMD) [5], [8]. Lack of a consistent association between FMD and CD4 cell count suggests that immune status is not directly related to endothelial dysfunction [5], [8], [9], . In a large cohort study, there was also no association between CD4 cell count and risk of myocardial infarction [12]. Despite careful study, the specific mediators of HIV-associated endothelial dysfunction have not been identified

CD4 T cell depletion in gut-associated lymphoid tissue (GALT) occurs within 4–6 weeks of primary HIV infection [13].Simian immunodeficiency virus-infected macaque models have demonstrated that gut bacteria are a source of circulating bacterial lipopolysaccharide (LPS) [14]. CD4 T-cell depletion in GALT coincides with increased expression of genes associated with inflammation and decreased expression of genes regulating epithelial barrier and digestive functions, mucosal repair and regeneration which suggests disruption of the gut microenvironment [13]. Increased levels of LPS that occur in patients with chronic progressive HIV infection decreased after 48 weeks of effective anti-retroviral therapy (ART) but did not normalize [15]. These increased levels of LPS are associated with persistent immune activation [15], so by extension persistent immune activation itself is another potential mechanism of HIV-associated endothelial dysfunction.

Gut microbial translocation leads to increased circulating levels of LPS and other microbial products. LPS activates endothelial cells via a distinct signaling pathway and this may directly influence cardiovascular disease pathogenesis [16], [17]. Soluble CD14 (sCD14) is a circulating glycoprotein that binds LPS, subsequently allowing the interaction of LPS with another signaling receptor. Cell associated CD14 is a multifunctional receptor, a glycoprotein expressed on the surface of monocytes, macrophages and neutrophils with a specificity for LPS and other bacterial wall derived components [16]. The presence of sCD14 is required for LPS-induced injury and activation of endothelial cells.

Our aim was to establish a relationship between microbial translocation measures and endothelial dysfunction in HIV-infected subjects in two well-characterized study populations [5], [8] which used FMD as a marker of endothelial function. As has been shown in recent studies, impaired FMD has been associated with worse long-term cardiovascular disease prognosis [2], [18]. A wide variety of inflammatory and vascular biomarkers were previously performed in both parent studies including high sensitivity C-reactive protein (hsCRP), lipids, lipoproteins, interleukin-6, adiponectin and vascular cell adhesion molecule-1 (VCAM-1) but none correlated with FMD.

## Methods

### Study subjects

Two distinct cohorts were examined. ACTG 5152s [5] was a substudy of ACTG 5142 [19] a prospective, multicenter, randomized clinical trial of ART initiation that examined endothelial function in HIV-infected, treatment-naïve subjects randomly assigned to 1 of 3 ART regimens and followed over 24 weeks. Exclusion criteria for A5152s included prior use of ART, known cardiovascular disease, diabetes mellitus or use of lipid-lowering medications [5]. The study was approved by the institutional review board at participating AIDS Clinical Trials Group sites.

The second study [8] was a single site study performed at Indiana University which enrolled unselected subjects from local primary HIV care clinics. Exclusion criteria, similar to 5152s included known cardiovascular disease, diabetes mellitus, active opportunistic infections, uncontrolled hypertension or renal insufficiency. This study was approved by the Indiana University institutional review board. In both studies, subjects consented to having blood specimens saved for possible future analyses.

The primary outcome measure was endothelial function, assessed by ultrasonic measurement of brachial artery FMD 60 seconds after lower forearm cuff release expressed as a percentage change, as previously described [5], [8]. FMD exams from both studies were performed by technicians trained in the same laboratory using the same techniques and reading software.

### Assays

Plasma samples preserved with EDTA were stored at −70°C until the time of assay. LPS was quantified using the Endpoint Chromogenic LAL assay (Lonza, Basel, Switzerland) and sCD14 measurements were performed with the Quantikine ELISA from R&D systems (Minneapolis, MN) according to the manufacturer instructions. For both assays all samples were analyzed in triplicate.

### Analyses

Subjects who had FMD, LPS and sCD14 measurements available were included for analysis. Whole group and pre-specified sub-group analyses included 5152s subjects at baseline and week 24 as well as the Indiana subjects as a whole, in addition to analysis of subgroups on ART and not on ART. Other subgroups considered were the combination of Indiana subjects on ART plus the 5152s subjects at week 24 (all ART-treated subjects), Indiana subjects not on ART plus the ART naive baseline 5152s subjects (all subjects not on ART). Indiana ART-treated subjects with undetectable viral load (defined as ≤400 copies/mL) were analyzed alone as well as combined with those 5152s patients with undetectable viral load at week 24 (all subjects with undetectable viral load).

Pearson correlation coefficients were used to characterize the correlation between brachial FMD and LPS and sCD14. Paired T test was used to compare baseline and 24-week measures for the A5152s subjects. Linear regression was used to identify the independent contribution of LPS to the variation in brachial FMD controlling for gender, baseline brachial artery diameter, heart rate, systolic blood pressure and ART use. Because other inflammatory, lipid and HIV disease variables did not influence FMD in the parent studies [5], [8] these were not considered in these analyses. Two-sided p-value of <0.05 was considered statistically significant. Analyses were not corrected for multiple comparisons in this exploratory study. All analyses were performed using SAS 9.2 (SAS Inc., Cary, NC).

## Results

### Subject Characteristics

There were 75 subjects from ACTG 5152s and 85 subjects from the Indiana University study that were included for a total of 160 patients in the primary analysis.The mean age of the A5152s subjects was 35 years with 92% male (Table 1). The median CD4 count was 251 cells/mm^3^ at baseline; it increased to 391 cells/mm^3^ at week 24. After 24 weeks, 92% had undetectable HIV RNA. There was a significant increase in LPS levels at 24 weeks compared to baseline (32 pg/mL vs. 40 pg/mL, p = 0.02) but no change in sCD14. As previously reported baseline brachial artery diameter in the ACTG 5152s groups was 0.44 cm.

**Table 1:** Subject Characteristics.

	A5152s Baseline N = 75	A5152s Week 24 N = 67	Indiana on ART N = 46	Indiana not on ART N = 39
Age, years	35±8	35±8	40±8	38±11
Male sex, %	92	94	63	79
Race/Ethnicity %				
White, not Hispanic	55	58	63	56
Black	25	25	33	41
White, Hispanic	15	12	2	0
Other	5	5	2	3
Time on ART	–	24 weeks	40 months	–
CD4, median (interquartile range)	251 (129,369)	391 (272,560)	464 (362,761)	322 (216,432)
Log_10_ HIV RNA	4.9±0.6	1.9±0.6	2.9±0.7	4.2±1.1
% HIV RNA <400 copies/mL	0	92	84	11
sCD14, 10^6^ pg/mL	2.14±.62	2.0±.64	1.72±.40	1.72±.47
LPS, pg/mL	32±21	40±32*	33±24	26±16
% FMD, median (interquartile range)	4.02 (1.98, 5.51)	5.46 (3.35, 6.78)	5.21 (2.25, 7.12)	5.19 (2.50, 6.90)

Values are means (±standard deviation) except where noted. ART – antiretroviral therapy. sCD14 – soluble CD14. LPS – lipopolysaccharide. FMD - Flow mediated dilation.

* p = 0.02 for the difference between baseline and week 24 in ACTG 5152s subjects.

The Indiana study was a cross-sectional study that included subjects on ART(n = 46) (n = 46) as well as those not on ART (n = 39) with the latter including both ART naïve and previously experienced subjects. The mean age was 40 years for those on ART and 38 years for those not on ART. For those on ART, the median CD4 count was 464 cells/mm^3^; 322 cells/mm^3^ for those not on ART. For individuals on ART, the proportion with undetectable HIV RNA was 84%; it was 11% for those not on ART. The median duration of ART in the treated group was 40 months. sCD14 levels were similar between groups. The mean LPS levels for those on ART was 33 pg/mL versus 26 pg/mL for those not on ART (p = 0.09). As previously reported [8] the Indiana cohort had a baseline brachial artery diameter of 0.40 cm which was similar in all groups.

### Correlations with markers of microbial translocation

There was no significant correlation between brachial FMD and either sCD14 or LPS in the group as a whole. There also was no correlation between FMD and markers of translocation in the ACTG group either at baseline or at week 24, nor in the subjects not on ART in the Indiana cohort (Table 2). Combining all of the untreated subjects (Indiana cohort not on ART plus the ACTG group at baseline) there also was no significant correlation of FMD with either LPS or sCD14 (data not shown). Among the Indiana cohort on ART there was a negative correlation between LPS and brachial FMD (r = −0.33, p = 0.02) but no correlation with sCD14 (Table 2). After limiting the analysis to only those Indiana subjects with undetectable viral load (N = 38) a similar, significant inverse correlation between LPS and endothelial function was observed (r = −0.36, p = 0.03), but there was no significant relationship between FMD and sCD14.

**Table 2:** Pearson Correlation Coefficients of Markers of Microbial Translocation with Brachial Artery Flow- Mediated Dilation.

	All subjects*	A5152s Baseline	A5152s Week 24	Indiana on ART	Indiana not on ART
N	160	75	67	46	39
sCD14	−0.15P = 0.07	−0.05P = 0.7	−0.09P = 0.5	−0.04P = 0.9	−0.23P = 0.2
LPS	−0.06P = 0.45	0.07P = 0.5	−0.2P = 0.2	**−0.33** **P = 0.02**	0.16P = 0.3

* Includes all subject data from baseline A5152s evaluations and all subjects in the Indiana cross-sectional study.

ART – antiretroviral therapy. sCD14 – soluble CD14. LPS – lipopolysaccharide. Significant correlations are shown in bold.

**Figure 1:**
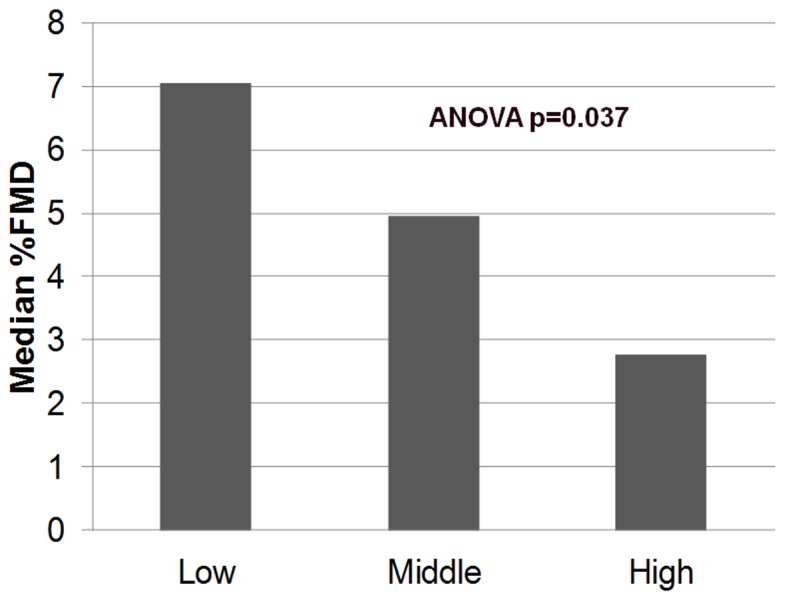
Median % flow mediated dilation by tertile of plasma lipopolysaccharide levels among ART treated subjects in the IU study.

When controlling for gender, baseline brachial artery diameter, heart rate, systolic blood pressure and ART use, LPS remained an independent predictor of FMD in those Indiana subjects on ART (p = 0.02). There was a progressive step-wise decrease in median % FMD across increasing tertiles of LPS levels (Figure 1) which was statistically significant (p = 0.037 by ANOVA). In the highest tertile of LPS levels (median 44.6 pg/ml [IQR 38.4, 62.8]), FMD was markedly impaired (median 2.76%).

## Discussion

In subjects on prolonged ART (mean of 40 months) we observed a statistically significant negative correlation between LPS levels and FMD, suggesting that increased microbial translocation is associated with endothelial dysfunction in chronically ART-treated subjects. A significant relationship between LPS levels and FMD was not seen in those individuals not on ART or in those on short-term (24 weeks) ART. Soluble CD14 levels were not significantly correlated with FMD in any analyses. However, LPS is directly involved in the causal pathway of endothelial dysfunction [16], [17] and thus may be a superior marker for this effect than a marker of monocyte activation such as sCD14 which does not directly activate endothelial cells. We speculate that the effect of LPS on endothelial function was seen only among those on longer term ART because it may require a longer duration of ART for the effects of persistent microbial translocation to be detected without the influence of other HIV-related disease complications and their associated inflammation. Further studies will need to be completed in order to make any definitive conclusions. LPS levels did not decrease and unexpectedly increased after 24 weeks of ART in the ACTG subjects, which may be an insufficient duration of treatment to detect reduced microbial translocation as was reported by Brenchley [14] who studied subjects after 48 weeks of ART.

Most [20], [21], [22], [23], but not all [24], [25], studies have shown that microbial translocation is greater with more advanced HIV infection. Chronic, low-level increases in circulating LPS represent a major pro-inflammatory mediator of atherosclerosis [26]. Increased levels of circulating LPS were a major risk factor for carotid atherosclerosis and have been associated with a variety of chronic bacterial infections in the general population [27]. Chronic infections conferred increased risk of atherosclerosis development even in low-risk subjects free of conventional cardiovascular disease risk factors [27]. Our results suggest that microbial translocation likely contributes to the increased cardiovascular disease risk in chronic treated HIV infection.

Limitations of this study include the relatively small number of subjects in the various subgroups, including those on prolonged ART. The cross-sectional nature of the Indiana study precludes observation of changes over time. The short duration of ART in the ACTG 5152s subjects may have prevented detection of a relationship between FMD and microbial translocation that may have occurred with a longer duration of treatment. Finally, differences in FMD may not necessarily translate into lesser long-term cardiovascular disease risk.

We conclude that in HIV-infected individuals on prolonged ART, greater degrees of microbial translocation, as reflected by higher circulating LPS levels, are associated with worse endothelial function. Because persistent microbial translocation may contribute to the increased cardiovascular disease risk observed in individuals on long-term ART, measures to reduce microbial translocation in HIV-infected patients may be warranted as an intervention to reduce chronic disease risk.
